# Comparing the impact of social support on the life satisfaction of widowed and non-widowed elders

**DOI:** 10.3389/fpsyg.2022.1060217

**Published:** 2022-11-17

**Authors:** Hua Tian, Jie Chen

**Affiliations:** ^1^College of Life Science, Xinyang Normal University, Xinyang, China; ^2^School of Marxism, Xinyang Normal University, Xinyang, China

**Keywords:** life satisfaction, social support, widowed elders, informal social support, formal social support

## Abstract

**Aim:**

To compare differences in life satisfaction between widowed and non-widowed elders based on social support.

**Methods:**

A total of 4,560 widowed and 3,655 non-widowed elders were selected from the Chinese Longitudinal Healthy Longevity Survey (CLHLS-2018). Ordinal logistic regression models and t-tests were performed using SPSS v20.

**Results:**

Both widowed and non-widowed elders had high levels of life satisfaction. Personal characteristics had a significant impact on the life satisfaction of both widowed and non-widowed elders. Endowment insurance, social trust, residence, self-rated health, and living with family had a significant impact on the life satisfaction of widowed elders (*p* < 0.001), while endowment insurance, government subsidy, and self-rated health significantly impacted non-widowed elders (*p* < 0.001). Self-rated health had the greatest impact on the life satisfaction of widowed and non-widowed elders (OR = 4.62/4.45), followed by endowment insurance (OR = 1.24/1.32).

**Conclusion:**

Social support can significantly improve life satisfaction, but its impact is heterogeneous. Informal social support plays a greater role in improving the life satisfaction of widowed elders, but formal social support plays a greater role in the life satisfaction of non-widowed elders.

## Introduction

There were approximately 350 million widowed elders in the world, of whom about 80% were widows (Sasson and Umberson, 2014). The spouse mortality rate of married couples increases with age (Yang et al., 2022). China is entering the rapid development stage of population aging, a significant component of which is an increasing widowhood rate year over year. There were an estimated 47.74 million widowed elderly people in China according to the sixth census, accounting for 26.89% of the total population (Cheng et al., 2019). China’s elderly widowed population is expected to reach 118.4 million by 2050 (Xiang et al., 2021). Widowhood is a disastrous event for the surviving partner (Tian and Chen, 2022). Widowhood has a serious impact on the physical, economic and emotional health of elders (Srivastava et al., 2021), and reduces overall life satisfaction (Yang et al., 2022). The life satisfaction of elders is an important indicator of life happiness. It is the cognitive component of subjective well-being (Diener et al., 2017), which is an important part of realizing healthy aging and implementing the healthy China strategy. Widows are more likely to receive social support from their children than widowers (Umberson et al., 1992). Social support has been shown to have a positive impact on the life satisfaction of elders (Li, 2004), but widowed elders have less social support (Stewart et al., 2001; Plaud and Urien, 2021). Social support is a subjective measure of the actual or perceived availability of resources from others, including emotional support and/or resource access (finances, goods, services, or information; Fiorillo and Sabatini, 2015). A systematic review of the relationship between widowhood and health in the elderly wrote that, “elderly widows and widowers manage their emotional pain through social ties, family support or the support of friends” (Holm et al., 2019). Previous works found that increased social support correlated with higher levels of life satisfaction in the elderly.

This study used CLHLS-2018 data to establish ordinal logistic regression models to explore the relationship between the social support and life satisfaction of widowed and non-widowed elders, based on formal social support and informal social support. It also sought to identify differences in this relationship, in order to deeply understand the differences in demand for elderly care services of widowed elders as special groups, permitting the effective use of family and social resources in order to improve life quality.

### Life satisfaction

Life satisfaction is an individual’s subjective evaluation of his environment and future life. It is an important component required for individuals to live a happy and peaceful life (Pan et al., 2019). It not only reflects the individual’s material and spiritual life, but also reflects the comparison between the individual’s actual situation and expected life. It is the theoretical basis for China’s desire to achieve healthy aging and implement the healthy China strategy (Deng and Tang, 2021). The factors that affect the life satisfaction of elders primarily include health status (Pan et al., 2022; Tian and Chen, 2022), economic level (Schwarz et al., 2010), pension mode (Zhou et al., 2017), social support (Tang et al., 2022), and information and communication technology (ICT; Li et al., 2022), with significant population differences (Williams et al., 2016). Life satisfaction is the basic dimension for measuring personal quality of life and subjective well-being (Fastame, 2021). Elder life satisfaction is associated with positive life outcomes such as health (Tian et al., 2022) and longevity (Ozdemir et al., 2022). Elders with good self-rated health are more likely to have a higher level of life satisfaction. This discovery was supported by research from six European countries, Russia, South Korea, Nepal, Turkey and Zambia (Mekonnen et al., 2022). Widowhood often has a negative impact on life satisfaction (Naess et al., 2015). The life satisfaction of unmarried or widowed people is lower than that of people with partners (Frasquilho et al., 2017), and people with low life satisfaction are often more prone to depression (Mei et al., 2021). Participating in social networks (Suanet and Antonucci, 2017; Cheng et al., 2022) such as keeping in touch with friends and maintaining an active lifestyle, often helps elders to regain life satisfaction (Wilder, 2016). The financial support and life care of children can significantly improve the life satisfaction of the elderly (Cui et al., 2022). Life satisfaction may also be affected by the elder’s number of children. Elders with more children are more likely to enjoy higher life quality and improved health (Yang et al., 2022). To a certain extent, the subjective well-being of the elderly increases with the number of children, and with an increased number of children comes a decline in, subjective well-being (Leng and Chen, 2019).

### Social support

The impact of social support on the health of the elderly has been a topic of widespread concern in social science and medicine (Feng et al., 2015). Social support is defined as a feeling or experience that a person is loved, cared for, respected and valued by others. Social support is a part of the social network of mutual assistance and obligation, which may involve a person clearly obtaining benefits from another person, or provide help and support (Litwin and Landau, 2000). Sources of social support range from intimate and stable relationships to more distant and unstable relationships (Chen et al., 2021). Social support includes objective, visible or practical support, such as direct material assistance, or the participation in social networks and groups involving family. It also includes subjective, empirical and emotional support, such as the emotional experience and satisfaction of individuals who are respected, supported and understood (Nazari et al., 2020). Zhang et al. (2022) believes that social support can be divided into emotional support, information support and companionship support from the perspective of function. Social support can also be divided into emotional support, such as affection, care, understanding, and substantive support, such as household and financial support, or into perceived or available support and actual support. Some works divide social support into four dimensions, namely formal social support, informal social support, subjective support and objective support (Ye and Su, 2022). Formal social support refers to the public service support provided by the government, institutions, communities, units and other formal organizations, such as medical insurance, endowment insurance and social assistance. Informal social support mainly refers to the social support network formed based on geographic and blood relations, including economic and material help from families, friends and neighbors, as well as emotional, behavioral and information support. Objective support mainly refers to the material assistance obtained by individuals, while subjective support refers to the emotional experience of individuals feeling respected, understood and supported. Social support has a positive impact on the life satisfaction of the elderly, because it can prevent loneliness and delay the development of ill health (Zhao et al., 2021; Thiel et al., 2022). The support provided by family members and friends is an important source of care and assistance for the elderly (Srivastava et al., 2021). Studies show that widowed elders have higher rates of negative emotions such as emptiness and loneliness, lower life satisfaction, higher mortality rates (Zebhauser et al., 2014) and greater need of subjective or informal support. Subjective support has a greater impact on the health of the elderly than other dimensions of objective and social support (Zhuori et al., 2019). At the same time, social support plays an important role in the life satisfaction of the elderly in Iran (Moeini et al., 2018), Japan (Kim et al., 1999), South Korea (Park and Song, 2012), and Saudi Arabia (Khusaifan and El Keshky, 2017) highlighting its importance in different cultural backgrounds.

## Materials and methods

### Participants

Participants were drawn from the Chinese Longitudinal Healthy Longevity Survey (CLHLS-2018), aged 65 and above. When screening for effective participants, ones that answered none or “I do not know” were eliminated. Respondents were asked to self-determine their marital status as (a) married and living with their spouse; (b) married, not living with their spouse; (c) divorced; (d) widowed; and (e) never married. After deleting the samples with missing and invalid variable values, 4,560 widowed elders and 3,655 non-widowed elders were selected. CLHLS-2018 was jointly conducted by the Chinese Center for Disease Control and Prevention and the Research Center for Healthy Aging and Development of the National Development Research Institute of Peking University. A total of 15,874 elders aged 65 and over from more than 500 investigations in 23 provinces (including rural and urban areas) of China participated in the questionnaire. The aim of this study was to evaluate the health status and influencing factors of elder life satisfaction, so as to identify potential health problems and provide information for scientific research, aging work and health policy.

### Study variables

#### Explained variable

Life satisfaction of the widowed and non-widowed elders was the explained variable. This variable reflects the self-evaluation and subjective feelings of the elderly about their current life. It is obtained through the question “how do you feel about your life now?.” Potential answers were very poor, poor, neutral, good and excellent, which were assigned values of 1, 2, 3, 4, and 5, respectively.

#### Control variables

Participants completed questions concerning their gender, registered permanent residence, residence, age, type of elderly care and self-rated health. Self-rated health data were obtained through the question “how do you feel about your own physical health now?” The answers “very good, good or neutral” were defined as good health, while “not good or very poor” were defined as poor health. Participants who answered “unable to answer or did not answer” were removed from the study.

#### Explanatory variables

Social support variables were the core explanatory variables. Social support variables were divided into formal social support and informal social support variables. Formal social support had four variables, endowment insurance, medical insurance, community service and government subsidy. Informal social support mainly include child support and social interactions. Support of children was examined using the three dimensions of child economic support, children’s sick care, and children’s emotional support, using the following three questions: “what is your main source of life now?” “Who will take care of you when you are sick or sick?” and “Who do you usually chat with most?” The answers “son, daughter, daughter-in-law, son-in-law, grandchildren or their spouses” were defined as having child support, while any other answer was no support. Social interactions were studied based on the three dimensions of “social trust, outdoor activities and social activities,” using the following three questions from the CLHLS-2018 questionnaire, “Do you often feel that people around you are untrustworthy?” “How often do you visit and socialize with friends?” and “How often do you participate in organized social activities?” The answers “always, often or sometimes” were defined as having social interactions, with all other answers defined as none.

### Data analysis

SPSS v20 (IBM, Armonk, NY, United States) was used for data analysis. Descriptive statistics, such as percentage, frequency, mean, standard deviation, t-test and ordinal logistic regression were used to reveal the influence of social support on the life satisfaction of widowed elders. During regression analyses, a regression relationship between one or more covariates and one dependent variable is encountered. When the dependent variable is an ordinal classification variable, an ordinal regression analysis can be applied. Ordinal logistic regression, also known as hierarchical regression, can model the dependence of a polytomous ordinal response on a set of predictive variables (factor variables or covariates). The research object in this study was the life satisfaction of the widowed and non-widowed elders, which is an ordinal discrete variable divided into five grades: poor, poor, neutral, good, and excellent. This can thus be estimated using an ordinal logistic regression model. The model is a multivariate discrete selection model that takes the logical distribution as the probability distribution of random error terms.

The logit equation is as follows:


$$\begin{array}{l} \mathrm{logit}\left(p_{ij}\right)=\ln\left[\frac{p_{i\left(y\leq j|x\right)}}{1-p_{i\left(y\leq j|x\right)}}\right]\\ \;=\alpha_{j}+\left(\beta_{1}x_{i1}+\beta_{2}x_{i2}+\cdots +\beta_{p}x_{ip}\right)+\;\varepsilon_{i,j} \end{array}$$

$$\left(i=1,\;2,\;\cdots \mathrm{,n;}\;j=1,\;2,\;3,\;4,\;5\right)$$

In the above formula, y represents life satisfaction, and j represents the assignment of life satisfaction (j=1, 2, 3, 4, 5). i represents the ith sample. $p_{i,j}$represents the cumulative probability of the first j values of theith sample. x represent the explanatory and control variables that affect the life satisfaction of the widowed and non-widowed elderly. ε is the random error term. α is a constant term. β is the variable coefficient.

## Results

### Demographic characteristics

Based on Tables 1, 2, widowed elders have high levels of life satisfaction, with 19.85% very satisfied with life, and 50.90% are satisfied with life, yielding a total proportion of very satisfied and satisfied elders of is 70.75%. For non-widowed elders, the proportion of very satisfied and satisfied with life was 68.29%, slightly lower than that of widowed elders.

**Table 1:** Variables definition.

Characteristic variables		Variables definition
Dependent variable		
	Life satisfaction	1 = Very poor, 2 = Poor, 3 = Neutral, 4 = Good, 5 = Excellent
Control variables		
	Gender	1 = Male, 2 = Female
	Registered permanent residence	1 = Urban, 2 = Rural
	Residence	1 = City, 2 = Town, 3 = Rural
	Age	True age
	Self-rated health	0 = No, 1 = Yes
	Type of elderly care	1 = Living with family, 2 = living alone, 3 = Institutional care
Formal social support		
	Endowment insurance	0 = No, 1 = Yes
	Medical insurance	0 = No, 1 = Yes
	Community service	0 = No, 1 = Yes
	Government subsidy	0 = No, 1 = Yes
Informal social support		
	Children’s economic support	0 = No, 1 = Yes
	Children’s sick care	0 = No, 1 = Yes
	Children’s emotional support	0 = No, 1 = Yes
	Social trust	0 = No, 1 = Yes
	Outdoor activities	0 = No, 1 = Yes
	Social activities	0 = No, 1 = Yes

**Table 2:** Descriptive statistics.

Characteristics		Widowed elderly (*n* = 4,560, %)		Non-widowed elderly (*n* = 3,655, %)	
Life satisfaction					
	Very poor	21	0.46	13	0.36
	Poor	131	2.87	96	2.63
	Neutral	1,182	25.92	1,050	28.73
	Good	2,321	50.90	1,686	46.13
	Excellent	905	19.85	810	22.16
Gender					
	Male	1,320	28.95	2,214	60.57
	Female	3,240	71.05	1,441	39.43
Registered permanent residence					
	Urban	764	16.75	531	14.53
	Rural	3,796	83.25	3,124	85.47
Residence					
	City	571	12.52	387	10.59
	Urban	2,347	51.47	1,340	36.66
	Rural	1,642	36.01	1,928	52.75
Age					
	65–79	793	17.39	2,333	63.83
	80–99	2,625	57.57	1,273	34.83
	≥100	1,142	25.04	49	1.34
Type of elderly care					
	Living with family	3,106	68.11	3,446	94.28
	Living alone	149	3.27	164	4.49
	Institution care	1,305	28.62	45	1.23
Self-rated health					
	Unhealthy	630	13.82	499	13.65
	Healthy	3,930	86.18	3,156	86.35

Of the 4,560 widowed elders, 71.05% were widows and 83.25% had a rural registered permanent residence, while 51.47 and 36.01% lived in urban and rural area, respectively. Over half (57.57%) of widowed elders were aged 80–99. 68.11% lived with their families and 86.18% self-reported that they were healthy. Widowed elders were mainly female (71.05%), while non-widowed elders were mainly male (60.57%). All other variables were equivalent between non-widowed and widowed elders.

### Comparison of variables between widowed and non-widowed elders

Based on Tables 3, 4 the average life satisfaction of widowed and non-widowed elders was 2.13, with no statistical difference between the two groups (*p* = 0.856). With respect to formal social support, only the government subsidy variable was equivalent between widowed and non-widowed elders (*p* = 0.031), with the other three variables significantly different (*p* < 0.001). The variable averages of medical insurance and community service were high for both widowed and non-widowed elders, especially with respect to medical insurance. The averages and standard deviations of the two groups were 0.90 ± 0.298 and 0.88 ± 0.327, respectively.

**Table 3:** Differences in social support between widowed and non-widowed elders.

Variables	Items	Widowed elders (*n* = 4,560)	Non-widowed elders (*n* = 3,655)	*p* value
Dependent variable				
	Life satisfaction	2.13 ± 0.772	2.13 ± 0.793	0.856
Formal social support				
	Endowment insurance	0.33 ± 0.471	0.38 ± 0.485	*p* < 0.001
	Medical insurance	0.88 ± 0.327	0.90 ± 0.298	*p* < 0.001
	Community service	0.60 ± 0.490	0.62 ± 0.484	*p* < 0.001
	Government subsidy	0.13 ± 0.336	0.11 ± 0.312	0.031
Informal social support				
	Children’s economic support	0.66 ± 0.475	0.38 ± 0.487	*p* < 0.001
	Children’s sick care	0.93 ± 0.260	0.31 ± 0.461	*p* < 0.001
	Children’s emotional support	0.78 ± 0.414	0.11 ± 0.312	*p* < 0.001
	Social trust	0.14 ± 0.348	0.15 ± 0.352	0.627
	Outdoor activities	0.42 ± 0.493	0.55 ± 0.498	*p* < 0.001
	Social activities	0.03 ± 0.170	0.05 ± 0.221	*p* < 0.001

**Table 4:** Gender differences in the social support of widowed and non-widowed elders.

Items	Widowed elders (*n* = 4,560)			Non-widowed elders (*n* = 3,655)		
	Widowers (*n* = 1,320)	Widows (*n* = 3,240)	*P* value	Male (*n* = 2,214)	Female (*n* = 1,441)	*P* value
Endowment insurance	0.33 ± 0.469	0.35 ± 0.476	0.204	0.37 ± 0.482	0.39 ± 0.489	0.096
Medical insurance	0.87 ± 0.334	0.89 ± 0.309	*p* < 0.05	0.91 ± 0.290	0.89 ± 0.309	0.162
Community service	0.60 ± 0.489	0.59 ± 0.492	0.434	0.62 ± 0.486	0.64 ± 0.481	0.236
Government subsidy	0.14 ± 0.345	0.11 ± 0.314	*p* < 0.05	0.12 ± 0.327	0.09 ± 0.285	*p* < 0.001
Children’s economic support	0.68 ± 0.468	0.61 ± 0.488	*p* < 0.001	0.38 ± 0.485	0.39 ± 0.488	0.458
Children’s sick care	0.93 ± 0.257	0.92 ± 0.267	0.459	0.28 ± 0.447	0.35 ± 0.478	*p* < 0.001
Children’s emotional support	0.79 ± 0.405	0.75 ± 0.432	*p* < 0.05	0.08 ± 0.278	0.15 ± 0.355	*p* < 0.001
Social trust	0.14 ± 0.346	0.15 ± 0.355	0.421	0.14 ± 0.347	0.15 ± 0.360	0.292
Outdoor activities	0.41 ± 0.492	0.44 ± 0.497	0.068	0.55 ± 0.498	0.55 ± 0.498	0.790
Social activities	0.03 ± 0.171	0.03 ± 0.165	0.689	0.05 ± 0.226	0.05 ± 0.214	0.399

Informal social support includes two dimensions: children’s support and social interactions. As this work is about the impact of social support on the life satisfaction of widowed elders, spousal support is ignored, despite it being a very important dimension of informal social support. Specifically, there are extremely significant statistical differences between widowed and non-widowed elders in children’s economic support (*p* < 0.001), children’s sick care (*p* < 0.001), and children’s emotional support (*p* < 0.001), respectively. Moreover, the average of these three variables among widowed elders was higher than that of non-widowed elders. With respect to social interactions, there were statistically significant differences between widowed and non-widowed elders in outdoor activities (*p* < 0.001) and social activities (*p* < 0.001). The average of outdoor activities and social activity scores were low for both widowed and non-widowed elders. The widowed and non-widowed elders rarely participate in organized public social activities. Thus, while both the widowed and non-widowed elders had high life satisfaction, endowment insurance and government subsidies need to be improved in addition to, social trust and social activities, especially with respect to the child support of non-widowed elders.

In order to analyze gender differences in social support between widowed and non-widowed elderly, an independent sample *t*-test was used to compare formal and informal social support. For widowed elders (*n* = 4,560), only children’s economic support (*p* < 0.001) was significantly different between widowers and widows. Medical insurance (*p* < 0.05), government subsidy (*p* < 0.05) and children’s emotional support (*p* < 0.05) were also significantly different between widowers and widows. When the scores of widower and widow elders were combined, children’s support was better and social interactions were the worst in terms of utilized informal social support. In terms of formal social support, endowment insurance and government subsidies also need to be improved. For non-widowed elders (*n* = 3,655), all formal and informal social support variables of male and female non-widowed elders, perform poorly and need to be improved except for medical insurance and community services. In all, regardless of sex or widowed / non-widowed status, both medical insurance and community services perform well.

### Ordinal logistic regression of social support on the life satisfaction of widowed elders

As shown in Table 5, model 1 and model 2 include only formal social support variables and informal social support variables, while model 3 and 4 include all variables. In terms of formal social support, endowment insurance had a significant impact on the life satisfaction of widowed and non-widowed elders (*p* < 0.001). Of these, the odds ratios of endowment insurance variables were 1.24 and 1.32 (OR > 1), indicating that widowed and non-widowed elders with endowment insurance have higher life satisfaction than those without endowment insurance. The impact of endowment insurance on life satisfaction was greater among non-widowed than widowed elders. Of the formal social support variables, government subsidy was a statistically significant influence on the life satisfaction of non-widowed elders (*p* < 0.001), with an OR was 0.64. In other words, non-widowed elders with government subsidies were less likely to be satisfied with life than non-widowed elders without government subsidies. The influence of government subsidy variables on the life satisfaction of widowed elders was not significant.

**Table 5:** Impact of social support on the life satisfaction of widowed vs. non-widowed elders.

Variables		Widowed elderly (OR, *n* = 4,560)			Non-widowed elderly (OR, *n* = 3,655)
		Model 1	Model 2	Model 3	Model 4
Formal social support					
	Endowment insurance	1.28***		1.24***	1.32***
	Medical insurance	0.99		0.98	1.05
	Community service	1.08		1.08	1.14
	Government subsidy	0.97		0.89	0.64***
Informal social support					
	Children’s economic support		0.89	0.88	0.90
	Children’s sick care		1.13	1.14	0.90
	Children’s emotional support		1.18*	1.17*	0.96
	Social trust		1.52***	1.50***	1.02
	Outdoor activities		1.07	1.06	1.00
	Social activities		0.92	0.89	1.23
Control variables					
	Gender	0.94	0.94	0.93	0.89
	Registered permanent residence	1.53	1.53***	1.49***	1.33*
	Residence (City)	1.38*	1.38	1.37*	1.46**
	Residence (Town)	1.15*	1.16*	1.17*	0.97
	Age	1.01*	1.01	1.01*	1.00
	Self-rated health	4.59***	4.60***	4.62***	4.45***
	Type of elderly care (Family)	1.68***	1.07***	1.64***	1.14
	Type of elderly care (Alone)	1.25	1.46*	1.42*	0.86

Model 1–3 is the ordinal logistic regression of variables of widowed elders. Model 4 is the ordinal logistic regression of variables of non-widowed elders. The regression coefficient represents odd ratios. ***, ** and * represent significance levels of 0.1, 1, and 5%, respectively.

In terms of informal social support, social trust variable had a significant impact on the life satisfaction of widowed elders (*p* < 0.001), in addition to children’s emotional support (*p* < 0.05). Compared with other forms of informal social support, social trust had a greater impact on the life satisfaction of widowed elders (OR = 1.50). However, informal social support variables had no significant impact on the life satisfaction of non-widowed elders. The impact of children’s economic support on the life satisfaction of widowed and non-widowed elders was insignificant.

Beyond gender, other control variables had a significant impact on the life satisfaction of widowed elders (*p* < 0.05) included self-rated health, living with family and a registered permanent residence. Self-rated health had an extremely significant impact not only on the life satisfaction of widowed elders (OR = 4.62), but on the life satisfaction of non-widowed elders as well (OR = 4.45). Living in the city had a significant impact on the life satisfaction of widowed (*p* < 0.05) and non-widowed elders (*p* < 0.01). Personal characteristics had a greater impact on the life satisfaction of widowed and non-widowed elders.

In short, social trust, residence, self-rated health, and living with family had the greatest impact on the life satisfaction of widowed elders (*p* < 0.001), while endowment insurance, government subsidy and self-rated health had the greatest impact on the life satisfaction of non-widowed elders (*p* < 0.001), Registered permanent residence (*p* < 0.05) and living in the city (*p* < 0.01) had a lesser but significant on non-widowed elder life satisfaction. Compared with other variables, self-rated health had the greatest impact on the life satisfaction of widowed and non-widowed elders (OR = 4.62/4.45), followed by endowment insurance (OR = 1.24/1.32). In conclusion, social support can significantly improve the life satisfaction of the elders. Endowment insurance and social trust are the supporting tools that directly affect the life satisfaction of widowed elders. The OR of informal social support was generally greater than that of formal social support, indicating that informal social support plays a greater role in improving the life satisfaction of widowed elderly. In contrast, formal social support plays a greater role in improving the life satisfaction of non-widowed elders. Personal characteristics also have a greater impact on the life satisfaction of widowed vs. non-widowed elders.

## Discussion

This study used the data from CLHLS-2018 to show that widowed and non-widowed elders had equivalently high levels of life satisfaction. Among widowed elders, endowment insurance and social trust were important factors that affected life satisfaction. Informal social support played a greater role in improving the life satisfaction of widowed elders vs. non-widowed elders. However, formal social support played a greater role in non-widowed elder life satisfaction, in particular endowment insurance. In November 2019, the Central Committee of the Communist Party and the State Council initiated the National Medium and Long Term Plan for Actively Responding to the Aging of Population (the “Plan” for short), which shows that coping with population aging has become a national issue in China. The plan points out that we should steadily increase the endowment wealth reserve and consolidate the social wealth reserve to deal with the aging population. As a system that covers the entire population and ensures basic life support for retirees, consolidation of the wealth reserve of the basic endowment insurance is the way to achieve the steady growth of the endowment wealth reserve (Chen, 2022). Endowment insurance is the main source of income for the elderly (Han et al., 2022). The main economic support of rural widowed elders is from their children, with the pension playing a complementary role (Li, 2022). Economic independence is the cornerstone of the independence of both widowed and non-widowed elders, and improves their sense of security. The current endowment insurance system in China does not make special arrangements for widowed elders. Whether they are employees of government institutions or enterprises, the pension income level after retirement only depends on the individual’s performance in the labor market over the course of their life and the local average wage level. It is not linked to the spouse’s employment history, there is no survivor’s pension policy, and the coverage and security level of survivors’ subsidies and pensions are also very limited. When the spouse is alive, the husband and wife can share the economic resources of the family. Once the spouse dies, the widows or widowers who lack personal pension security can easily fall into poverty (Zhao and Zhang, 2019; Muhammad et al., 2021), especially if they lack economic support from their children. Therefore, endowment insurance and child economic support are complementary and integrated, regardless of widowed or non-widowed status.

Social trust is an important aspect of social capital (Murgas et al., 2022). Trust is a kind of expectation for the behavior and performance of people based on social interaction and personal experience. It has complexity and variability, and different trust models can be formed under different social and cultural backgrounds and personal growth backgrounds (Li and Liang, 2002). Paldam (2000) believed that social trust includes general trust (trust unspecified people) and special trust (trust known people or specific institutions). Leung et al. (2011) found that generalized interpersonal trust and institutional trust are independently related to well-being, although there is only a weakly positive correlation between these two kinds of trust. Zhang and Zhang (2015) believed that a high degree of trust in public institutions will lead to or enhance the elderly’s belief in social and world justice and fairness, thereby further improving life satisfaction. In China, the social interactions of elders are mostly characterized by circle distributions. The core circle is the family life circle, followed by the close friend circle, and the neighbor or stranger circle as the outermost layer. The degree of social trust of the elderly towards different groups is different. Studies have shown that trust in family members, friends and neighbors has a significant positive impact on the emotional health, subjective well-being and social status of the elderly in rural China (Chen and Zhu, 2021). Social trust has also been positively correlated with life satisfaction. The social trust of rural elders is worse than that of urban elders. The special trust level of the elderly is significantly higher than the general trust level. The more the elder participates in social activities, the higher their life satisfaction (Gao et al., 2015). Social support from friends plays an important role in preventing and regulating anxiety among rural elders (Zhao et al., 2022).

There are several limitations to this study that should be acknowledged. Firstly, only self-rated health was used as a health variable. Self-rated health reflects both objective and subjective aspects of health status, serving as a comprehensive reflection of physical and mental health, and an important and reliable predictor of health outcomes (such as disability, incidence rate and mortality) in healthy elders. Secondly, this was only a cross-sectional study of the life satisfaction of widowed and non-widowed elders. Changes in life satisfaction over time and the impact of family and community environment variables on life satisfaction were not considered. Despite these limitations, our findings provide valuable insight into the heterogeneous roles that informal and formal social support play in improving the life satisfaction of widowed and non-widowed elders.

## Conclusion

This work aimed to evaluate the impact of social support on the life satisfaction of widowed and non-widowed elders. Regardless of sex or widowed vs. non-widowed status, both medical insurance and self-rated health had a strong influence on life satisfaction. Further, support from children had a stronger influence on the life satisfaction of widowed elders than on non-widowed elders perhaps due to the loss of the spouse’s accompany. Among non-widowed elders, endowment insurance had a positive impact on life satisfaction, while government subsidies had a negative impact. Fortunately, both male and female widowed and non-widowed elders had high levels of life satisfaction. In conclusion, social support can significantly improve the life satisfaction of widowed and non-widowed elders, but had a heterogeneous impact on these groups. While informal social support played a greater role in improving the life satisfaction of widowed elders, formal social support played a greater role among non-widowed elders.

## Data availability statement

The raw data supporting the conclusions of this article will be made available by the authors, without undue reservation.

## Author contributions

HT and JC designed this study together. HT performed the statistical analysis and drafted the manuscript. JC revised the manuscript. Both authors contributed to the article and approved the submitted version.

## Funding

This work was supported by Nanhu Scholars Program for Young Scholars of XYNU (Xinyang Normal University, China).

## Conflict of interest

The authors declare that the research was conducted in the absence of any commercial or financial relationships that could be construed as a potential conflict of interest.

## Publisher’s note

All claims expressed in this article are solely those of the authors and do not necessarily represent those of their affiliated organizations, or those of the publisher, the editors and the reviewers. Any product that may be evaluated in this article, or claim that may be made by its manufacturer, is not guaranteed or endorsed by the publisher.
